# Supplementary figures and images for: Crosstalk Between Omental Adipose-Derived Stem Cells and Gastric Cancer Cells Regulates Cancer Stemness and Chemotherapy Resistance
Source: Cancers (Basel). 2024 Dec 23;16(24):4275. doi: 10.3390/cancers16244275 (PMC11674675; doi:10.3390/cancers16244275)

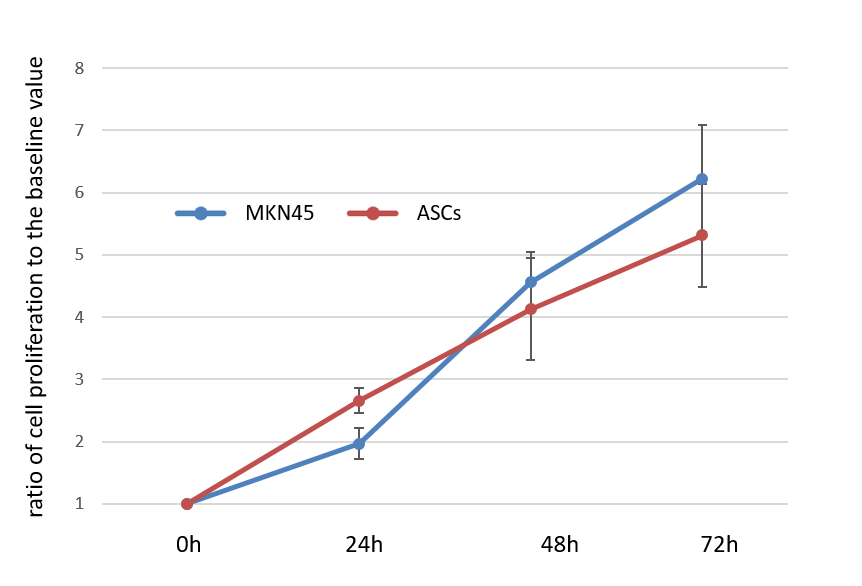

Supplement: Supplementary file 1 [file cancers-16-04275-s001.zip › Figure S1.png]

Figure S2 Original blots for Figure 5B

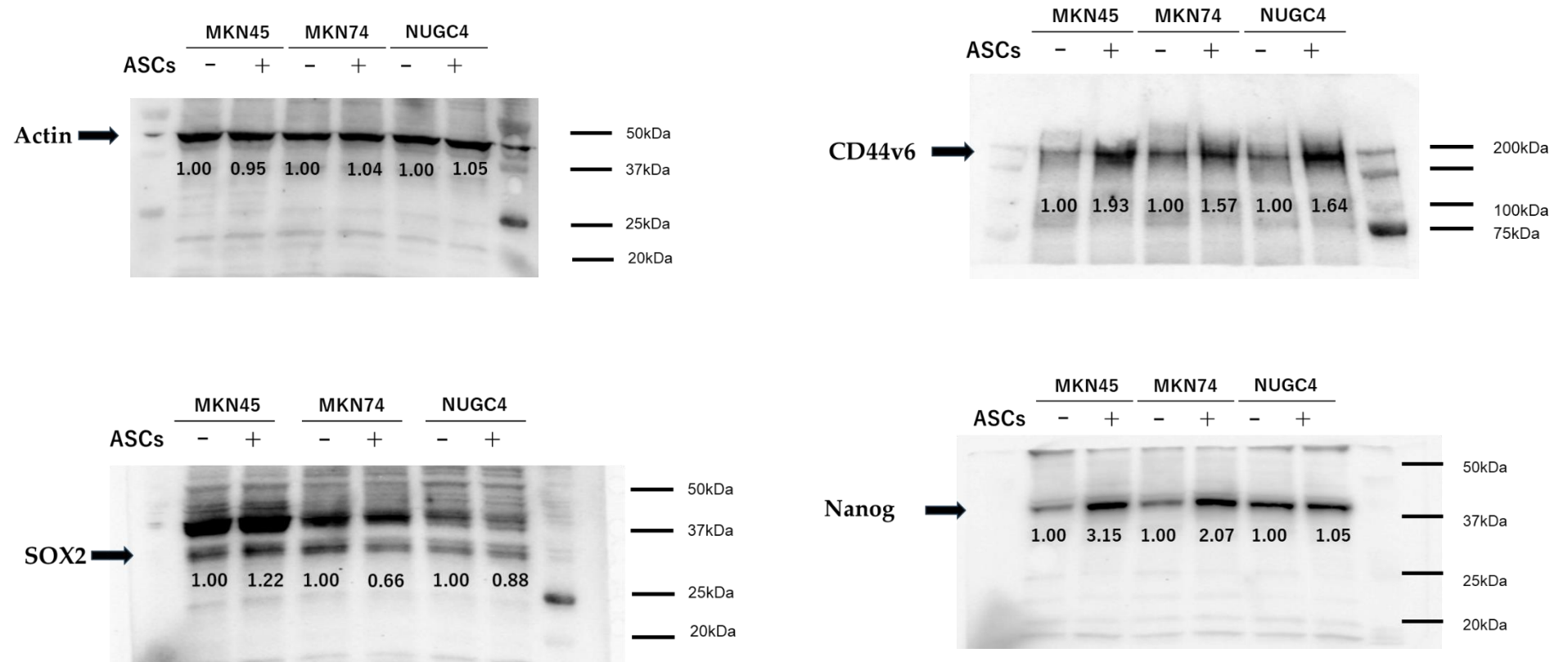

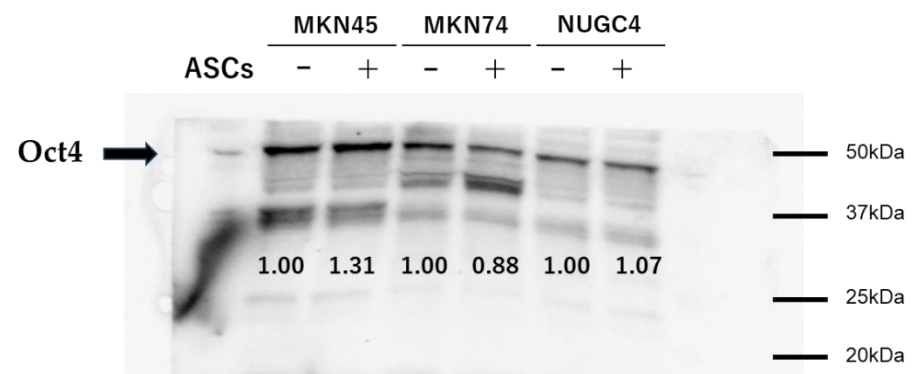

Figure S2 Original blots for Figure 6B

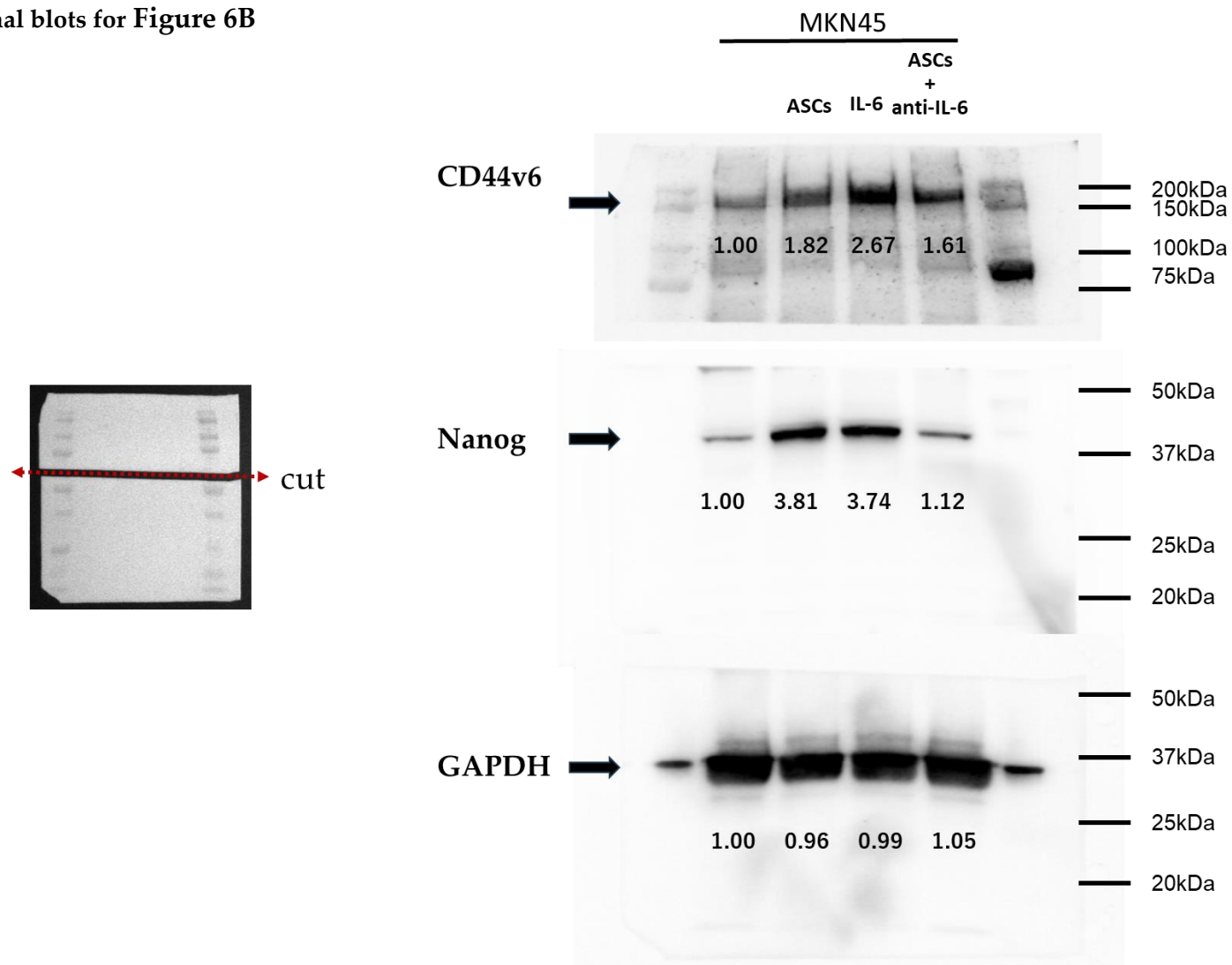

Supplement: Supplementary file 1 [file cancers-16-04275-s001.zip › Figure S2.pdf]
